# Neighborhood socioeconomic inequality based on everyday mobility predicts COVID-19 infection in San Francisco, Seattle, and Wisconsin

**DOI:** 10.1126/sciadv.abl3825

**Published:** 2022-02-18

**Authors:** Brian L. Levy, Karl Vachuska, S. V. Subramanian, Robert J. Sampson

**Affiliations:** 1 Department of Sociology and Anthropology, George Mason University, Fairfax, VA 22030, USA.; 2 Center for Social Science Research, George Mason University, Fairfax, VA 22030, USA.; 3 Department of Sociology, University of Wisconsin-Madison, Madison, WI 53706, USA.; 4 Department of Social and Behavioral Sciences, Harvard T.H. Chan School of Public Health, Boston, MA 02115, USA.; 5 Department of Sociology, Harvard University, Cambridge, MA 02138, USA.; 6 Harvard Center for Population and Development Studies, Cambridge, MA 02138, USA.

## Abstract

Race and class disparities in COVID-19 cases are well documented, but pathways of possible transmission by neighborhood inequality are not. This study uses administrative data on COVID-19 cases for roughly 2000 census tracts in Wisconsin, Seattle/King County, and San Francisco to analyze how neighborhood socioeconomic (dis)advantage predicts cumulative caseloads through February 2021. Unlike past research, we measure a neighborhood’s disadvantage level using both its residents’ demographics and the demographics of neighborhoods its residents visit and are visited by, leveraging daily mobility data from 45 million mobile devices. In all three jurisdictions, we find sizable disparities in COVID-19 caseloads. Disadvantage in a neighborhood’s mobility network has greater impact than its residents’ socioeconomic characteristics. We also find disparities by neighborhood racial/ethnic composition, which can be explained, in part, by residential and mobility-based disadvantage. Neighborhood conditions measured before a pandemic offer substantial predictive power for subsequent incidence, with mobility-based disadvantage playing an important role.

## Teaser

Disadvantage in a neighborhood’s mobility network has a larger impact than its own economic status on COVID-19 incidence.

## INTRODUCTION

The coronavirus disease 2019 (COVID-19) pandemic exposed the depth of health inequality in the United States, with racial and ethnic minorities and lower-income populations much more likely to contract the disease (*1*, *2*). These patterns are consistent with broader and long-standing health inequalities in the United States (*3*). Although neighborhood conditions are an important driver of health inequalities (*4*), to date, research has focused mainly on descriptive variations among larger geographic units, such as county, city, or zip-code COVID-19 caseloads [e.g., (*1*, *5*, *6*)].

In this study, we analyze COVID-19 incidence at the fine-grained neighborhood level. Research on health disparities and residential segregation (*7*) is extensive, motivated, in part, by the enduring fact of racial segregation in the United States (*8*). Recent decades also had increases in income-based segregation, particularly among families (*9*, *10*). Such patterns of residential segregation correlate with inequalities across a range of neighborhood-based resources that affect health, including availability and quality of healthcare services; exposure to toxins, pollution, or violence; quality of infrastructure; location of food deserts; and a broad range of factors promoting upward economic mobility (*11*–*14*).

In their everyday lives, however, individuals move through many neighborhoods as they go about their daily rounds (*15*). Homophily in mobility patterns can reinforce the isolation of affluence or disadvantage (*16*). Among the 50 largest U.S. cities, residents of primarily Black and Hispanic neighborhoods—whether poor or not—are far less likely to visit either nonpoor or white middle-class neighborhoods than residents of primarily white neighborhoods (*17*). Mobility patterns also have the potential to form structural connections between neighborhoods with independent consequences for the well-being of their residents. One study finds that adjusting for residential disadvantage, disadvantage in a neighborhood’s mobility-based network has added value in explaining neighborhood rates of violence; this indicates three distinct types of socioeconomic (dis)advantage a neighborhood can experience, or potential triple disadvantage (*18*). The first, residential neighborhood disadvantage (RND), is based on the socioeconomic characteristics of its residents. The other two are based on the average characteristics of the neighborhoods its residents tend to visit and receive visits from.

(Dis)advantage in a neighborhood’s mobility network could affect its COVID-19 incidence through individual or institutional pathways, although analysis of these is beyond the scope of this study. At the individual level, mobility connections between neighborhoods present the opportunity for pandemic transmission (*6*, *19*). COVID-19 is transmitted primarily by airborne pathways, and lockdowns or requests to stay at home are a recognition that by limiting exposure to human contacts induced by travel across geographic space, the risk of transmission is reduced (*20*). Mobility travel declined markedly in 2020 after the pandemic escalated (*21*). Given the greater incidence of COVID-19 and reduced capacity for distancing in economically disadvantaged communities (*1*, *22*), high rates of mobility between disadvantaged neighborhoods potentially increase transmission risk.

At the institutional level, durable neighborhood ties can influence public or private investment and the allocation of scarce resources (*23*–*25*). For example, early in the pandemic, access to COVID-19 testing evinced disparities by a zip code’s race and class (*26*). Similar disparities exist in zip code vaccination rate (*27*). Social resources like collective efficacy, which facilitates behavior regulation and capacity to act toward a shared goal (*28*), also promote individual and neighborhood well-being during crisis (*29*, *30*). In the context of COVID-19, we expect that collective efficacy would increase enforcement of norms around social distancing, masking, and virus testing. Residential poverty and instability are negatively correlated with collective efficacy (*23*, *28*), and recent analysis finds that disadvantage in a neighborhood’s mobility network increases interpersonal friction, potentially further reducing collective efficacy (*18*).

Here, we take a “triple disadvantage” perspective and hypothesize that socioeconomic disadvantage among a neighborhood’s residents, as well as the neighborhoods they visit and are visited by, will affect COVID-19 infection levels (*18*). The first of these, RND, is the domain of traditional neighborhood effects research. The latter two, which we combine as mobility-based neighborhood disadvantage (MND), are the subject of nascent scholarship. Because there can be threshold effects in both neighborhood effects (*31*, *32*) and in disease spread (*33*), we expect associations between neighborhood disadvantage (ND) and COVID-19 infections to be nonlinear. High levels of residential or mobility-based advantage may be especially protective, whereas high disadvantage in one or both contexts may be especially detrimental.

### Data

To analyze neighborhood-level inequality in COVID-19 infection, we integrate data from three source types: SafeGraph’s “Social Distance Metrics” Dataset, the 2015–2019 American Community Survey (ACS), and administrative data on COVID-19 test results for 1977 census tracts in three locations (the state of Wisconsin, Seattle/King County, and San Francisco). These three jurisdictions are the only major U.S. geographic locations for which we found well-documented COVID-19 case data at the neighborhood level. They also represent qualitatively different contexts in several ways. Wisconsin displays sizable variation in urban or rural land use and is politically moderate, although it has relatively low levels of racial and ethnic diversity compared to other U.S. geographies. San Francisco and King County have greater levels of racial and ethnic diversity, and San Francisco is entirely urban, whereas King County is a mix of urban and suburban. Both are also politically more progressive. Our analytic sample reflects all tracts with complete data for all variables. This includes 1390 of 1409 tracts in Wisconsin, all 397 tracts in King County, and 190 of 197 tracts in San Francisco. Of the excluded tracts, 20 of 26 have zero or minimal resident population and cover water or parks. The remaining six excluded tracts lack sufficient data to calculate all measures of residential neighborhood conditions, primarily due to low or unique populations.

Our dependent variable is the count of residents in a neighborhood that tested positive for COVID-19 by the end of February 2021. We focus on cumulative cases through February because this window of observation captures essentially all of the winter surge in cases across the United States, while avoiding the period from March 2021 onward when vaccine access was becoming widely available, which could fundamentally alter the relationships of interest. Across all tracts in our sample, we observe 661,612 residents testing positive. Counts and incidence are much higher in Wisconsin. Mean COVID-19–positive cases per 1000 resident population is 94.5 across all Wisconsin tracts, whereas it is 38.6 and 36.7 across the San Francisco and King County tracts, respectively. The variation in COVID-19 incidence is another benefit to studying three locations, as it allows us to test the significance of ND in contexts of relatively high and low infection.

Our primary independent variables are measures of RND and MND. Consistent with past research, RND is the principal factor from a factor analysis of seven measures of residents’ socioeconomic status using nationwide tract-level ACS data. Following Levy *et al.* (*18*), we calculate two measures of MND using a valued digraph of interneighborhood mobility patterns measured with 2019 SafeGraph data. Outdegree neighborhood disadvantage (OND) is the weighted average RND level of the other neighborhoods visited, often many times, by a neighborhood’s residents. Indegree neighborhood disadvantage (IND) is the weighted average RND level of neighborhoods from which a neighborhood receives visits. OND and IND are very highly correlated, with *R* ranging from 0.87 to 0.97 over our three locations. Extending the approach of Levy *et al.* (*18*), we thus average OND and IND for our measure of MND. We measure MND using 2019 mobility data to avoid endogeneity to the pandemic and identify more permanent structural connections. Still, MND values are nearly perfectly correlated between 2019 and 2020—despite sizable declines in mobility—further reinforcing the structural nature of interneighborhood mobility networks (see fig. S1). Measuring MND before the pandemic allows us to test its predictive capacity as an “ecometric” (*34*).

Mean RND in Wisconsin is nearly equivalent to the nationwide average, whereas mean RND levels are relatively low in San Francisco and King County. Mean MND in Wisconsin is also similar to the nation, whereas mean levels are lower in the two western counties. The absence of disadvantage is a form of privilege, and we look at both ends of the distribution to detect independent associations of mobility-based advantage and disadvantage. Table S1 presents summary statistics for all variables. Materials and Methods provides further information about our analytic approach.

**Fig. 1.**
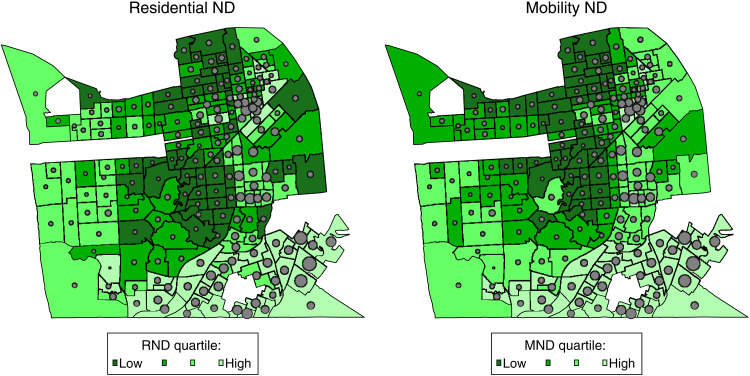
Map of San Francisco neighborhood proportions COVID-19 positive by RND and MND quartiles. Note that COVID-19 cases per 1000 resident population range from 4.4 to 182.5. Dots are scaled proportionally to a tract’s incidence.

To illustrate the nature of spatial inequality in our measures, Fig. 1 presents maps of the levels of RND and MND along with a scaled indicator of the incidence of COVID-19 cases per 1000 resident population in San Francisco. The top quartiles of both RND and MND are concentrated in the southeastern corner of the city, which is also where the highest rates of COVID-19 infection are seen. The lowest rates of COVID-19 infection generally appear in central San Francisco where the lowest levels of RND and MND cluster, although low MND is notably more tightly clustered. There is a general overlap between quartiles for RND and MND—roughly three-fifths match—but there is variation. For example, the Central Waterfront/Dogpatch neighborhood in eastern San Francisco is in the lowest quartile of RND but the second highest quartile of MND. Consistent with the latter, its COVID-19 infection rate is in the city’s 60th percentile.

### Analysis

We address two research questions in our analysis. First, what is the relationship between ND and COVID-19 incidence? We estimate a series of Poisson models with robust errors and controls for relevant observed characteristics (see Materials and Methods). Given the variation in land use and population characteristics of our three locations, we conduct separate analyses for each location. To allow for potential nonlinearity in focal relationships, we operationalize both RND and MND using location-specific indicator variables that identify observations in the lowest and highest quartiles of each ND measure, with the middle two quartiles serving as the reference group.

A primary aim of this first analysis is to assess whether RND and MND add predictive power beyond other theoretically relevant covariates. Here, we draw on the methodological concept of “ecometrics,” which aims to provide reliable, valid, and systematic metrics of a neighborhood’s context that can be regularly updated and used to monitor the neighborhood’s well-being (*34*, *35*). Accurate monitoring and prediction is especially important in response to a pandemic or other crisis. Although observational research like ours generally precludes estimation of a precisely identified causal effect, we do consider the extent to which estimated associations are robust to unobserved confounding using the E value sensitivity analysis (*36*, *37*). The myriad macro-level social structures and micro-level interactional processes that mediate and are mediated by neighborhood inequalities can be difficult to capture through an experiment—and experiments at the neighborhood level are relatively rare. To complement experiments, this paper presents the sort of careful observational research that is necessary to identify plausible ways in which neighborhoods affect well-being, in this case COVID-19 incidence (*23*, *38*).

Our second research question investigates the extent to which ND can explain neighborhood-level racial disparities in COVID-19 case counts. Black and Hispanic Americans are enduring outsized impacts from COVID-19, and both groups, though especially Black Americans, have experienced persistent inequity in neighborhoods, housing, and activity spaces due to racial segregation. We use the approach described by Valeri and VanderWeele (*39*) to investigate whether RND or MND mediates the relationships between a neighborhood’s percentages of Black and Hispanic residents and its COVID-19 cases. We estimate the total “effect” of neighborhood racial composition as the difference in adjusted predicted COVID-19 incidence between neighborhoods with low (5th percentile) and high (95th percentile) shares of a racial/ethnic group. This estimated total effect is not, of course, a race effect; rather, it is an estimated impact of the many long-standing aspects of racism in the U.S. net of other covariates. Given the lack of overlap in racial demographics between Milwaukee and the rest of Wisconsin (see Materials and Methods), we estimate separate models for Milwaukee County and all other Wisconsin tracts. We consider these models exploratory “mechanism sketch[es]” (*40*) of the potential role of ND in racial inequality.

### Limitations

We acknowledge two limitations to this study. First, given testing challenges and asymptomatic cases, observed COVID-19 case counts will be undercounts. We expect this to conservatively bias our estimates of hypothesized associations because undercounts are likely higher among traditionally disadvantaged groups (see also Materials and Methods). Second, our models control for many important covariates, but unobserved confounding remains a potential source of bias. We use the E value to calculate how robust our estimated relationships between ND and COVID-19 incidence are to potential confounders. More generally, observational research is an important tool for identifying, analyzing, and intervening to address neighborhood-based inequalities (*23*).

## RESULTS

### ND and COVID-19 incidence

Our baseline model of tract COVID-19 counts in Wisconsin (Table 1, model 0) adjusts only for residential population and provides a benchmark against which we can judge the added value of the ND measures and controls. Our four indicators for RND or MND (model 1) have explanatory power that is 35 to 45% the size of all controls combined (model 2), based on all three measures of fit and predictive power—Bayesian information criterion (BIC), pseudo-*R*^2^, and root mean square error (RMSE). Once we adjust for controls, the added explanatory value of MND (model 4) is roughly four to five times that of RND (model 3). The reduction in RMSE from adding MND to the controls-only model is more than one-fifth of the reduction in RMSE achieved by adding all controls to the baseline model. This indicates the substantive importance of MND in explaining Wisconsin COVID-19 cases. In Wisconsin, the benefits associated with low RND or MND are greater than the potential harm of high ND. Including both RND and MND (model 5), MND remains salient. The concentration of mobility-based advantage, and likely a wealth of correlated resources, is an independent protective factor against COVID-19 infection.

**Table 1.** Poisson models of cumulative tract COVID-19–positive cases through February 2021 (Wisconsin). Note that controls include tract-level log population density, age composition (shares 0 to 4, 5 to 17, 18 to 24, and 65+), share living in a household with four or more members, mean household size, share living in group quarters, shares of workers in four sectors (health services, food preparation/service, personal care, and production), share of workers that carpool, share of workers that take public transit, share Black, and share Hispanic. County fixed effects (F.E.) are dummy variables separately identifying each county with at least 30 tracts. Robust SEs are in brackets. *N* = 1390 for all models. ***P ≤ 0.001, **P ≤ 0.01, *P ≤ 0.05, ^†^*P* ≤ 0.1.

	**Model 0**	**Model 1**	**Model 2**	**Model 3**	**Model 4**	**Model 5**	**Model 6**	**Model 7**
RND low		−0.030		−0.074^***^		−0.024	−0.051^*^	−0.053^*^
		[0.019]		[0.022]		[0.021]	[0.021]	[0.021]
RND high		0.110^***^		0.043		0.041	0.047^†^	0.055^*^
		[0.025]		[0.028]		[0.027]	[0.026]	[0.026]
MND low		−0.143^***^			−0.144^***^	−0.136^***^	−0.136^***^	−0.141^***^
		[0.019]			[0.018]	[0.019]	[0.027]	[0.025]
MND high		−0.059^*^			−0.040^†^	−0.047^*^	−0.029	−0.002
		[0.023]			[0.022]	[0.021]	[0.022]	[0.024]
Constant	−2.355^***^	−2.320^***^	−2.444^***^	−2.393^***^	−2.361^***^	−2.341^***^	−2.186^***^	−2.194^***^
	[0.008]	[0.012]	[0.139]	[0.136]	[0.136]	[0.135]	[0.131]	[0.131]
Offset: ln(pop.)	X	X	X	X	X	X	X	X
Main controls			X	X	X	X	X	X
County F.E.							X	X
Spatial lag RND								X
BIC	51,580	47,384	39,668	39,284	38,150	38,063	35,292	35,174
Pseudo-*R*^2^	0	0.0819	0.2332	0.2410	0.2629	0.2649	0.3199	0.3225
RMSE	124.1	116.5	106.5	105.8	102.7	102.6	97.9	97.7

Unobserved confounding is a concern with these models, particularly in a geographically diverse location like Wisconsin. To account for time-invariant county-level characteristics, we add county fixed effects in model 6. Our main model includes indicator variables for each of the nine counties with 30 or more tracts in an attempt to balance concerns with statistical power with those of county-level confounding; more than half of the 72 counties have 10 or fewer tracts. Results are substantively quite similar using alternative strategies—fixed effects for the largest three counties, counties with 50 or more tracts, or all counties (fig. S2). The significance and magnitude of the association between low MND and COVID-19 cases are unchanged. In addition, coefficients on low and high RND emerge as marginally significant, though still smaller in magnitude than low MND. We also explore whether spatial clustering of similar neighborhoods and propinquity effects explain the significance of MND by adding a spatial lag of RND as the average RND of all adjacent neighborhoods using a queen contiguity matrix (model 7). Again, the association between MND and COVID-19 is unchanged.

**Fig. 2.**
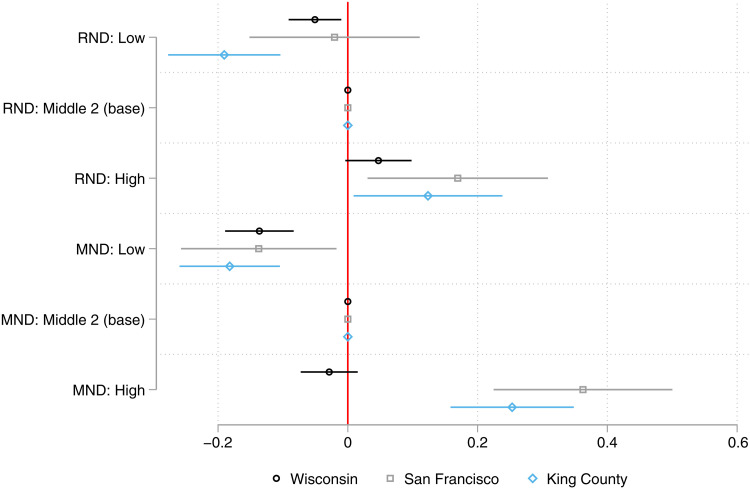
Parameter estimates for the adjusted association between ND indicators and COVID-19 case count by location. Note that coefficients are estimated in the main model for each location: Table 1, model 6 for Wisconsin; table S4, model 5 for San Francisco; and table S5, model 5 for King County. Error bars represent 95% confidence intervals.

Figure 2 graphs the coefficients on the ND variables and their 95% confidence intervals from our main model for Wisconsin, as well as the analogous models for San Francisco and King County. Across all three locations, disparities in COVID-19 cases by MND are larger than those by RND. In King County, all four indicators for low and high levels of RND and MND are significant in the expected direction. Three of the four indicators are significant in San Francisco. Magnitudes of the associations for each ND indicator are broadly similar across the jurisdictions with one clear exception. High MND is strongly associated with increased COVID-19 cases in San Francisco and King County, whereas it is not significantly associated with cases in Wisconsin. The cutoffs for classification in the top quartile of MND in San Francisco and King County (−0.72 and −0.29, respectively) are not far removed from the cutoff for the bottom quartile of MND in Wisconsin (−0.33). Thus, the observed pattern of MND associations across the three locations could reflect a particular benefit of overall affluence in the neighborhood network regardless of location-specific rank.

Measures of ND also yield appreciable improvement in explanatory power in San Francisco and King County (see the Supplementary Materials). In San Francisco, improvement in explanatory power associated with the ND measures alone is roughly 40 to 60% of the size of improvement with all controls combined. In King County, the explanatory power gains based on ND measures over baseline are essentially equivalent to those based on adding all controls. In both counties, we also find that MND provides greater explanatory power over a controls-only model than does RND.

**Fig. 3.**
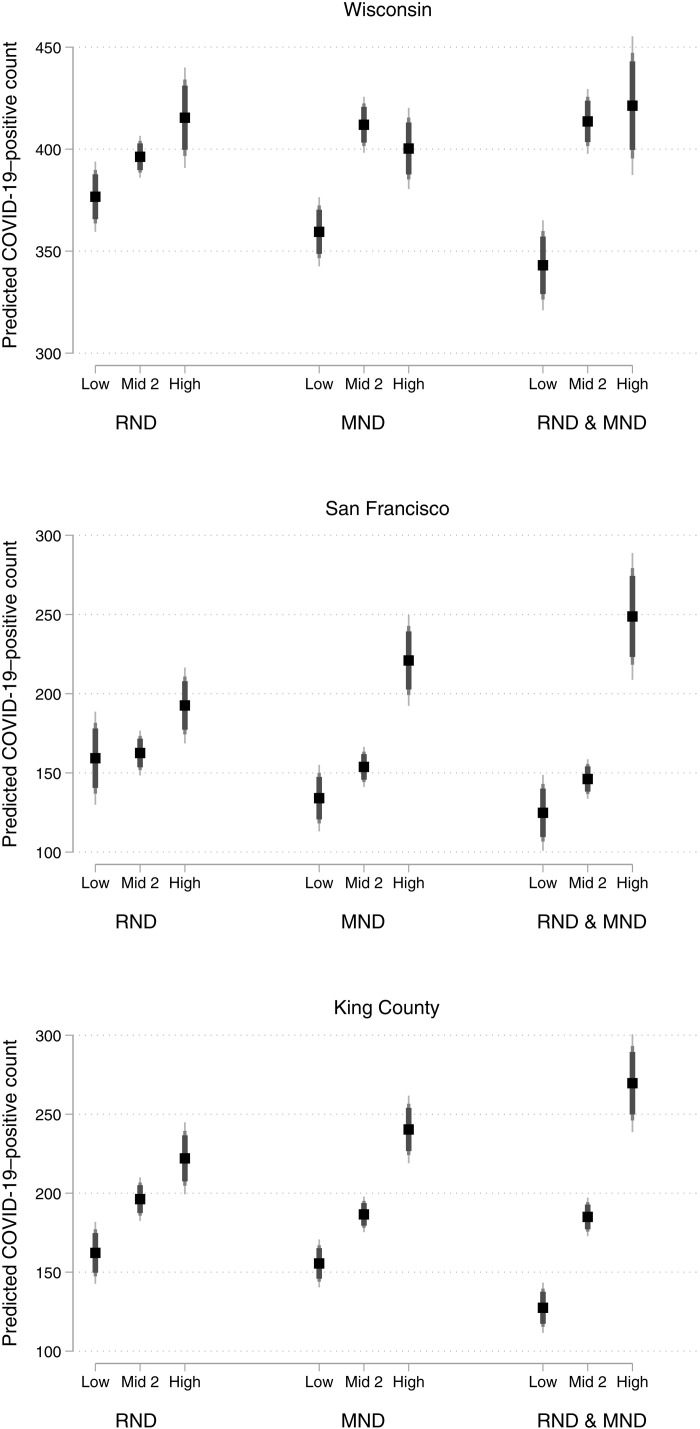
Average adjusted predicted COVID-19–positive cases by ND indicators from the main model for each location. Note that error bars represent 90, 95, and 99% confidence intervals. Predictions are based on the main model for each location and hold covariates at their observed values.

Figure 3 plots tract average adjusted predicted COVID-19 case counts based on values of the ND variables and estimates from our main models. For each location, the left cluster of plots is based only on RND values, the middle cluster is based only on MND values, and the right cluster is based on illustrative combinations of RND and MND. All covariates not used in a cluster are held at their observed values for predictions. In Wisconsin, looking at variation only in MND, middle and high MND tracts have predicted COVID-19 case counts that are 11 to 15% larger than low MND tracts, adjusting for all other covariates. Considering the joint impact of RND and MND, tracts with middle and high levels of ND on both measures have predicted counts that are 21 and 23% larger, respectively, than tracts with low ND values. As in Wisconsin, disparities in predicted counts based on MND in the two western counties are larger than those based on RND. When considering the joint impact of RND and MND, tracts with high levels on both metrics have predicted COVID-19 counts that are 99 and 112% larger than the counts of tracts with low levels of both ND types in San Francisco and King County, respectively. Whereas low levels of ND were especially protective in Wisconsin, high levels of ND are particularly detrimental in San Francisco. Low and high levels of both ND measures are of roughly similar importance for COVID-19 cases in King County.

We now calculate the *E* value to consider the extent to which these rate ratios of adjusted disparities in COVID-19 caseloads between neighborhoods with high levels of both RND and MND (“high ND”) versus those with low levels of both RND and MND (“low ND”) are robust to potential unobserved confounding. The *E*-value quantifies, on the risk ratio scale, the strength of association between unobserved confounder(s) and both COVID-19 incidence and the set of ND indicators that would be required to move the rate ratio to null (*36*, *37*). We consider both ND measures jointly because they often co-occur at the same level, and each is independently predictive of COVID-19 incidence.

In Wisconsin, the 23% increase in COVID-19 caseloads associated with high ND versus low ND could be explained entirely by an unobserved confounder(s) associated with COVID-19 incidence and the set of ND measures at a risk ratio of 1.76 each (Table 2). Weaker confounding would not yield a null rate ratio. Given the wide range of controls included in our model, an unobserved confounder with a risk ratio of 1.76 might be unlikely, but it is certainly plausible that an unknown factor could explain the complete disparity associated with high versus low ND. The 95% confidence interval of the rate ratio could be moved to include the null with an unobserved confounder associated with both dependent and set of ND variables at a risk ratio of 1.45.

**Table 2.** E-value sensitivity analysis for the main model in each jurisdiction. Note that adjusted predicted COVID-19 caseloads in Wisconsin, San Francisco, and King County are based on estimates from Table 1 (model 6), table S4 (model 5), and table S5 (model 5), respectively.

	**Adj.** **predicted** **COVID-19** **caseload** **with 95%** **confidence** **interval**	**Rate ratio**		
		**Estimate**	**95% confidence interval**	
Wisconsin				
Low RND, low MND	343.1 [326.3, 359.8]			
High RND, high MND	421.3 [395.4, 447.2]			
High-low ND rate ratio		1.228	1.108	1.348
*E* value		1.76	1.45	
San Francisco				
Low RND, low MND	124.8 [106.6, 143.0]			
High RND, high MND	248.8 [218.3, 279.2]			
High-low ND rate ratio		1.993	1.516	2.470
*E* value		3.40	2.40	
King County				
Low RND, low MND	127.5 [115.4, 139.5]			
High RND, high MND	269.6 [246.1, 293.2]			
High-low ND rate ratio		2.116	1.766	2.465
*E* value		3.65	2.93	

Much stronger omitted confounding is needed to explain the COVID-19 disparities by ND in the western counties. In San Francisco, an unobserved confounder would need to be associated with both COVID-19 incidence and the set of ND variables at a risk ratio of 3.4 each to completely explain the doubling in COVID-19 incidence associated with high versus low ND. The lower tail of the rate ratio’s 95% confidence interval could be moved to include the null by a confounder associated with both dependent variable and ND at a risk ratio of 2.4 each. In King County, the risk ratio for the association between confounder and both COVID-19 incidence and ND required to explain the complete disparity in COVID-19 by ND or move its confidence interval to include the null is 3.65 and 2.93, respectively. In both San Francisco and King County, these are sizable degrees of omitted confounding, roughly double or more that of Wisconsin. Overall, our results indicate that unobserved confounding would have to be moderately strong (Wisconsin) to quite large (Seattle and San Francisco) to explain away the associations.

### Racial disparities in COVID-19 and ND

**Fig. 4.**
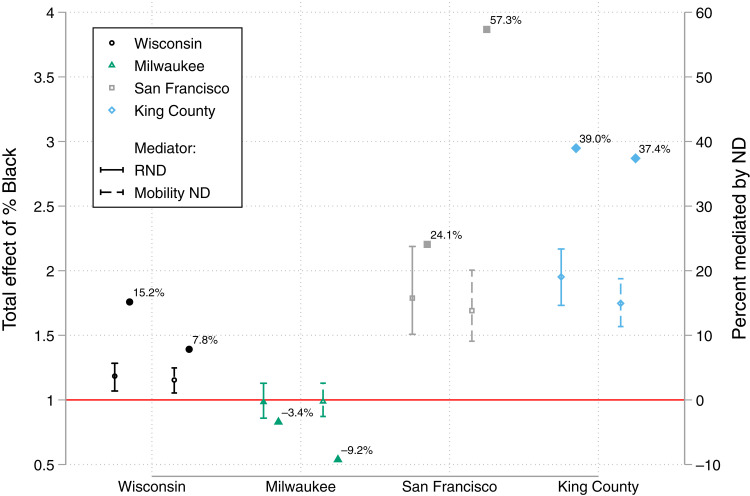
Mediation of the relationship between tract percent Black and COVID-19 caseload by RND and MND. Note that full results from mediation models appear in the Supplementary Materials. Estimated total effects are on the risk ratio scale and based on location-specific 5th and 95th percentiles of tract proportion Black. For Wisconsin (excluding Milwaukee), Milwaukee, San Francisco, and King County, those contrasts are 0 versus 0.122, 0.005 versus 0.915, 0 versus 0.179, and 0 versus 0.240, respectively.

Complete results for all mediation models of COVID-19 disparities by neighborhood racial/ethnic composition appear in the Supplementary Materials. In Wisconsin (excluding Milwaukee), neighborhoods with a relatively high share of Black residents were associated with a roughly 18.5% increase in COVID-19–positive risk versus neighborhoods with low share of Black residents (*P* < 0.05; Fig. 4). RND can mediate approximately 15.2% of the estimated total effect associated with neighborhood racial composition. Estimated total effects within a location are generally quite consistent regardless of the mediator. For Wisconsin, the second mediation model indicates that MND is a somewhat weaker mediator, explaining only 7.8% of the estimated total effect associated with share Black. In Milwaukee County, percent Black has an estimated null total effect on COVID-19 risk, rendering mediation estimates moot.

The two diverse western counties both demonstrate much stronger COVID-19 disparities by neighborhood percent Black and greater mediation by the ND variables. The estimated total effect associated with high versus low percent Black in San Francisco and in King County ranges from a 69 to 95% increase in neighborhood population risk of contracting COVID-19. In San Francisco, almost three-fifths of this risk increase can be explained by variation in MND levels. RND is still a fairly strong potential mediator, though substantively much weaker than MND. In King County, RND and MND have similar mediation potential for the risk increase associated with high share Black, explaining nearly two-fifths of the estimated total effect.

**Fig. 5.**
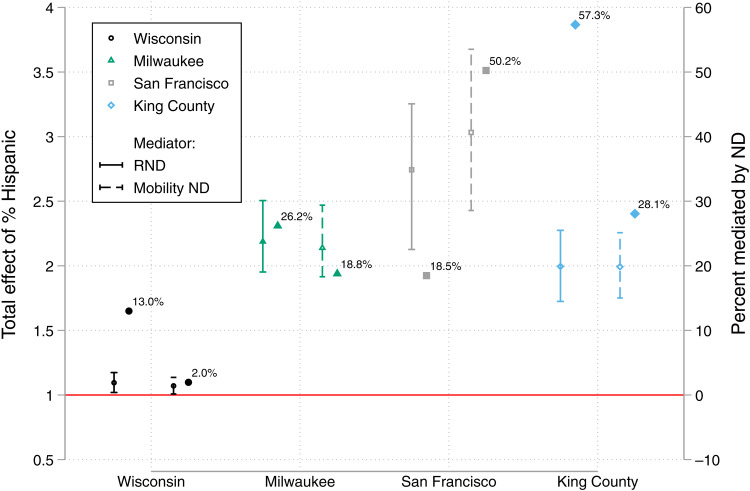
Mediation of the relationship between tract percent Hispanic and COVID-19 caseload by RND and MND. Note that full results from mediation models appear in the Supplementary Materials. Estimated total effects are on the risk ratio scale and based on location-specific 5th and 95th percentiles of tract proportion Hispanic. For Wisconsin (excluding Milwaukee), Milwaukee, San Francisco, and King County, those contrasts are 0.004 versus 0.169, 0.007 versus 0.729, 0.042 versus 0.397, and 0.021 versus 0.268, respectively.

The estimated total effect of high versus low percent Hispanic in Wisconsin (excluding Milwaukee) is a nearly 10% increase in COVID-19–positive risk (*P* < 0.05; Fig. 5). RND can mediate a small portion of this modest increase. Estimated total effects of share Hispanic are substantially larger in Milwaukee County, San Francisco, and King County—somewhere between a doubling and tripling of COVID-19 risk depending on the location. In Milwaukee, roughly one-fifth to one-quarter of this increase can be mediated by one of the ND measures. In San Francisco, over half of the tripling in neighborhood COVID-19 risk associated with high percent Hispanic can be explained by variation in MND levels. In King County, nearly three-fifths of the doubling in neighborhood COVID-19 risk related to high versus low share Hispanic can be explained by RND.

## DISCUSSION

Our analysis documents sizable neighborhood disparities in COVID-19 caseloads that are tied to residents’ mobility patterns by neighborhood socioeconomic status. As expected, RND is associated with significant increases in COVID-19 incidence in separate analyses of three qualitatively different locations: the state of Wisconsin; San Francisco, CA; and King County, WA. In line with recently developed theory about the importance of structural connections between neighborhoods based on residents’ mobility patterns (*18*, *23*), however, we find that ND in a neighborhood’s network of neighborhoods its residents visit and receive visits from is more predictive for COVID-19 incidence than its own level of RND. We also note evidence that overall affluence in the neighborhood network is especially protective. Considering both RND and MND, neighborhoods that are high on both have appreciably higher levels of COVID-19 than those that are low on both—sometimes twice as high. We further show the ability for ND to mediate sizable disparities in COVID-19 risk associated with neighborhood racial and ethnic composition. We are limited in our ability to draw conclusions about the causal pathways between ND and COVID-19 incidence given the observational nature of our research. We nonetheless addressed potential confounding by including a broad range of controls as well as county fixed effects in Wisconsin. Especially in San Francisco and King County, the results indicate that unobserved confounding would have to be quite large to explain away the associations.

Our results have public health and policy implications. Adjusting for many relevant characteristics, we document sizable variation in COVID-19 risk at the fine-grained neighborhood level for three distinct geographic locations. Moreover, RND and MND evince strong added value in explaining variation in COVID-19 incidence. Thus, triple ND could serve as a useful ecometric tool (*34*, *35*) that can be regularly updated and used to identify neighborhoods at risk of particularly adverse impacts during future pandemics or other crises. This previously unidentified form of mobility-based neighborhood inequity aligns with a longer history of health disparities and access to health care resources at the neighborhood level (*7*, *13*). Our findings indicate the importance of continued investment in and partnership with socioeconomically disadvantaged communities to facilitate care, as well as provide evidence for planning efforts to address ongoing inequality during the COVID-19 pandemic and responses to future pandemics or health events.

We also provide fresh evidence on how neighborhood-based disparities in an airborne pandemic arise. In line with recent models demonstrating the salience of everyday individual mobility patterns for pandemic spread (*6*), we find that ND in mobility-based connections between neighborhoods is more predictive of a neighborhood’s COVID-19 caseload than its own level of ND. Recent work demonstrates a similar pattern for a categorically different public health outcome: homicide (*18*). With growing attention to social determinants of health and associated spatial health disparities, connections between neighborhoods forged by residents’ mobility patterns represent a unique source of inequality.

## MATERIALS AND METHODS

We conduct parallel analyses of census tracts, a commonly used definition of neighborhoods, for Wisconsin, King County in Washington, and San Francisco County by merging data from three source types. Our dependent variable is the total count of each tract’s residents that have tested positive for COVID-19 through 28 February 2021. In Wisconsin and King County, all positive diagnostic test results confirming the presence of the virus causing COVID-19 are reported to the state’s Department of Health (*41*, *42*). Using home address data for individuals testing positive, Wisconsin and King County then aggregate and report counts of residents testing positive for the first time in each census tract. Duplicate positive tests or second infections are purged from the total counts in both locations. In October 2021, Wisconsin released an updated version of their COVID-19 database. Unlike the original data that we used in our analysis, the updated data count multiple positive tests for the same individual as unique positive cases. Despite this fact, our dependent variable from the original database is nearly perfectly correlated with the cumulative count on 28 February 2021 in the updated database. In San Francisco, total tract case counts are based on positive laboratory tests of residents reported to the county’s Department of Public Health (*43*). The Department conducts additional data verification or interview procedures to determine residence of individuals. Case totals represent new cases rather than a simple accounting of tests, but it is possible that an individual could be infected, recover, and later be reinfected, thus appearing twice in the data. This would be quite rare through February 2021. In all three jurisdictions, cumulative total cases will be undercounts given scarcity of confirmatory tests early in the pandemic, reluctance to be tested among some individuals, and the presence of asymptomatic cases for which individuals may not get tested (*44*). We do not expect the last of these to cause substantial bias in our estimates of relative risk between neighborhoods. To the extent that the first two yield bias, we expect that socioeconomically disadvantaged neighborhoods would have greater levels of undercount (*45*), which could make our estimates of the magnitude of hypothesized associations conservative.

Our primary independent variables are RND and MND. We measure RND using nationwide data from the 2015–2019 ACS to conduct a principal factor analysis of seven neighborhood characteristics: percentages of poverty, unemployment, single-headed households, public assistance receipt, adults without a high school diploma, adults with a bachelor’s degree or higher, and workers who are managers or professionals. The variables load strongly onto one factor with high reliability, and nationwide, RND is a continuous variable with a mean of zero and SD of roughly one.

Calculating measures of MND requires not only RND scores of visited or visiting neighborhoods but also data on the strength of a mobility tie between neighborhoods. We draw our U.S. mobility patterns from SafeGraph, a company that aggregates anonymized, repeatedly measured location data from a nationally representative group of 45 million smartphone devices that is provided by Veraset. Researchers have recently used SafeGraph mobility data to study segregation in Milwaukee and mobility changes during COVID-19 (*6*, *46*). For this study, we rely on SafeGraph’s Social Distance Metrics Dataset, which provides daily data on how many residents (devices) of each census block group visit every other block group at least once in that day. Home location for a device is determined by SafeGraph using machine learning as the common nighttime (6 p.m. to 7 a.m.) location of the device. A visit is defined as a cluster of proximal location pings with duration longer than 1 min. Devices can count for up to one visit in each block group in a single day. We aggregate visit counts from the block group level to the census tract level (block groups nest perfectly). This allows the maximum number of daily visits to a tract for any device to be the total number of block groups in the tract. We expect any bias introduced by this aggregation to be minimal.

We begin by constructing a nationwide mobility network as a valued digraph comprising a set of nodes (census tracts), *N* = {*n*1, *n*2, …, *nN*}; a set of edges, *E* = {*e*1, *e*2, …, *eE*}; and a set of values, *V* = {*v*1, *v*2, …, *vV*} (*47*). Values *V*(*n_ij_*) represent the extent of mobility of residents of tract *i* to tract *j* over all days (*a*) in the sample. We approximate *V*(*n_ij_*) as the total number of day-visitors to tract *j* who reside in tract *i* for the time period analyzed. In mathematical terms

$$V(n_{\mathrm{ij}})=\sum_{a=1}^{D}t_{\mathrm{aij}}$$ (1)

where *t_aij_* is the number of visitors to *j* that reside in *i* on day *a*, and *D* is the number of days of data in the analysis. To remove cross-country tourist visits, which likely have much less, if any, effect on a neighborhood’s capacity to achieve its sociopolitical goals, we restrict all *n_ij_* edges to occur between tracts within the same commuting zone (CZ) or, for counties on the border between CZs, between tracts in its CZ or contiguous counties. We use all 365 days in 2019 for our mobility data to develop measures of MND that are proximal to the pandemic but not affected by the marked shifts in mobility patterns during 2020. In other words, this network reflects the more stable, structural ties between neighborhoods that can prove crucial for their vitality.

We use the valued adjacency matrix in Eq. 1 to calculate the weighted average disadvantage level of extra-local neighborhoods to which any neighborhood *n_i_* is structurally connected through its residents’ intra-CZ mobility patterns, or OND

$$\text{OND}=\sum_{j=1}^{N}(\frac{V(n_{\mathrm{ij}})}{V(n_{i})-V(n_{\mathrm{ii}})}*{\text{RND}}_{j}),i\neq j$$ (2)

Here, *V*(*n_i_*) represents total number of visits from tract *i*, *V*(*n_ii_*) represents visits within tract *i* (loops), and RND*_j_* represents the residential disadvantage score of the visited neighborhood.

Next, we calculate the weighted average disadvantage level of extra-local neighborhoods to which any neighborhood *n_i_* is structurally connected through the intra-CZ visits it receives from residents of other neighborhoods, or IND. Neighborhoods vary in population size and visit rates; we adjust for these aspects in our calculations, as shown in Eq. 3, where *P_j_* is the population of sending neighborhood *n_j_*

$$\text{IND}=\frac{\sum_{j=1}^{N}{\text{RND}}_{j}*V(n_{\mathrm{ji}})*P_{j}}{\sum_{j=1}^{N}V(n_{\mathrm{ji}})*P_{j}},i\neq j$$ (3)

As our overall measure of MND, we then average OND and IND.

As a check on potential bias introduced by aggregation from block groups to tracts, we also calculate block group IND, OND, and MND scores using nonaggregated SafeGraph data (i.e., at the block group level), coupled with RND scores at the census block group level. We then calculate alternative specifications of tract-level MND as the population-weighted average MND scores of all block groups in a tract. The correlation of MND calculated in this alternative manner with MND calculated as we specify above is 0.988. This very strong correlation indicates that any bias from aggregation would be negligible.

Our control variables for this study use the 2015–2019 ACS and are informed by both theoretical expectations and recent research. We use an offset of log total neighborhood residential population, and we include controls for neighborhood log population density, age composition (shares of residents that are ages 0 to 4, 5 to 17, 18 to 24, and 65+), share living in a household with four or more members, mean household size, share living in group quarters, shares of workers in each of four sectors (health services, food preparation or service, personal care, and production), share of workers that carpool, share of workers that take public transit, share Black, and share Hispanic. We access all ACS data from Social Explorer.

### Statistical analysis

Separately for Wisconsin, San Francisco, and King County, we analyze the relationships of RND and MND with neighborhood total COVID-19 case counts using a series of Poisson models with robust errors. Our main model is

$$\text{ln}(\mu_{i})=\beta_{0}+\beta_{1}{\text{RND}}_{\text{low}}+\beta_{2}{\text{RND}}_{\text{high}}+\beta_{3}{\text{MND}}_{\text{low}}+\beta_{4}{\text{MND}}_{\text{high}}+\beta_{x}x_{i}+\text{ln}({\text{pop}}_{i})$$ (4)

We predict COVID-19 case counts (μ) in neighborhood *i* using indicator variables for location-specific low and high quartiles of tract RND as well as low and high quartiles of tract MND. We also include our vector of control variables (*x_i_*) as well as an offset for log residential population. In Wisconsin, our main model also includes parameters for indicator variables identifying each county with 30 or more tracts (β*_i_*δ*_i_*). Before our main model, we also estimate a sequence of nested models to assess the relative predictive power of our measures of ND as well as compare the predictive power of our ND measures with our controls.

We use the E value sensitivity analysis (*36*, *37*) for our main model in each jurisdiction to gauge how robust our estimated relationships between ND and COVID-19 incidence are to potential unobserved confounding. The E value identifies the strength of association between an unobserved confounder, or set of confounders, and both treatment and outcome that would be required to completely explain the estimated treatment-outcome relationship. E values are directly calculable for rate ratios. We use the rate ratio for average adjusted predicted COVID-19 caseloads in high ND (both RND and MND) versus low ND (both RND and MND) neighborhoods, which is taken directly from estimates in the right cluster for each jurisdiction in Fig. 3.

Our mediation analyses use the approach described in depth by Valeri and VanderWeele (*39*). This method extends the Baron and Kenny (*48*) framework to Poisson regression and allows for treatment-mediator interaction effects on outcome. Despite using language like treatment, outcome, and direct or indirect effect for convenience, we consider these mediation models mechanism sketches. The approach can incorporate, at most, one treatment variable and one mediator variable per model. Thus, for each location, we estimate four models representing all combinations of the neighborhood race (share Black and share Hispanic) and disadvantage (RND and MND) variables. Our mediation models make two important departures from the main models. First, given the low level of overlap in racial demographics between Milwaukee County and the rest of Wisconsin, we separately analyze mediation for Milwaukee. Mean tract proportion Black is over 12 times as high in Milwaukee County, and more than two-thirds of all Black Wisconsin residents live in the county. Hispanic residents are somewhat more evenly distributed, but mean tract proportion Hispanic is still nearly three times as high in Milwaukee County. Second, race and ND have fairly strong correlations across the jurisdictions, and our indicator variables for ND quartiles suppress a nontrivial portion of the variables’ variations. To better assess the full mediation potential of RND and MND, we use their continuous specifications in these analyses. Our general mediation models take the following form

$$\text{ln}\{E(Y|A=a,M=m,C=c)\}=\theta_{0}+\theta_{1}a+\theta_{2}m+\theta_{3}\mathrm{am}+\theta_{c}c$$ (5)

Equation 5 is our outcome model where *Y* is COVID-19 case count, *A* is the neighborhood share Black (Hispanic) “treatment” variable, *M* is potential ND mediator, and *C* is a vector of controls, which includes log residential population and the neighborhood racial composition variable not being used as treatment. We then fit the linear model below for the mediator

$$E(M|A=a,C=c)=\beta_{0}+\beta_{1}a+\beta_{c}c$$ (6)

Analyzing mediation in this framework with Poisson models requires specifying two values (*a*, a*) for treatment to generate a predicted difference in COVID-19 counts. We use the location-specific 5th and 95th percentiles of neighborhood racial composition for this contrast. The natural direct effect (NDE) is on the risk ratio scale and calculated as

$$\text{NDE}=\text{exp}[\text{ln}\{\frac{E(Y_{{\mathrm{aM}}_{a^{*}}}|c)}{E(Y_{a^{*}M_{a^{*}}}|c)}\}]$$ (7)

The natural indirect effect (NIE) is also on the risk ratio scale and calculated as

$$\text{NIE}=\text{exp}[\text{ln}\{\frac{E(Y_{{\mathrm{aM}}_{a}}|c)}{E(Y_{{\mathrm{aM}}_{a^{*}}}|c)}\}]$$ (8)

The total effect of the treatment is then the product of NDE and NIE. We bootstrap 95% confidence intervals using 1000 replications for NDE, NIE, and total effect (TE) as recommended by Valeri and VanderWeele (*39*). The estimated proportion of the total effect mediated is

$$\mathrm{PM}=\frac{\text{NDE}*(\text{NIE}-1)}{\text{NDE}*\text{NIE}-1}$$ (9)

The key assumption for valid estimates in both our main and mediation models is no unobserved confounding. In the mediation models, this implies no unobserved confounding in three relationships: treatment to outcome, treatment to mediator, and mediator to outcome. We attempt to address this by including a range of theoretically relevant controls, as well as various county fixed effects strategies in Wisconsin. Although it is not possible to fully discount unobserved confounding, observational analyses are important in this context for several reasons. Among the most important is that governmental resources to combat the pandemic and its effects are often allocated spatially. Documenting low-level spatial inequalities can aid in targeting these efforts. Moreover, to the extent that measures of ND add value in explaining place-based disparities in COVID-19 caseloads, accounting for them will prove a useful addition for predictive models of pandemic spread that assist in combating the pandemic—even if ND is not the direct or distal cause of variation in COVID-19 caseloads. The results from our models of two large counties and one medium-sized state suggest that ND does add predictive value.
