# Supplementary material for: Neighborhood socioeconomic inequality based on everyday mobility predicts COVID-19 infection in San Francisco, Seattle, and Wisconsin
Source: Sci Adv. 2022 Feb 18;8(7):eabl3825. doi: 10.1126/sciadv.abl3825 (PMC8856620; doi:10.1126/sciadv.abl3825)
Supplement: Supplementary file 1 — Figs. S1 and S2 Tables S1 to S11 [file sciadv.abl3825_sm.pdf]

Science Advances
AAAS

Supplementary Materials for

Neighborhood socioeconomic inequality based on everyday mobility predicts COVID-19 infection in San Francisco, Seattle, and Wisconsin

Brian L. Levy*, Karl Vachuska, S. V. Subramanian, Robert J. Sampson

*Corresponding author. Email: blevy4@gmu.edu

Published 18 February 2022, *Sci. Adv.* **8**, eabl3825 (2022)
DOI: 10.1126/sciadv.abl3825

This PDF file includes:

Figs. S1 and S2
Tables S1 to S11

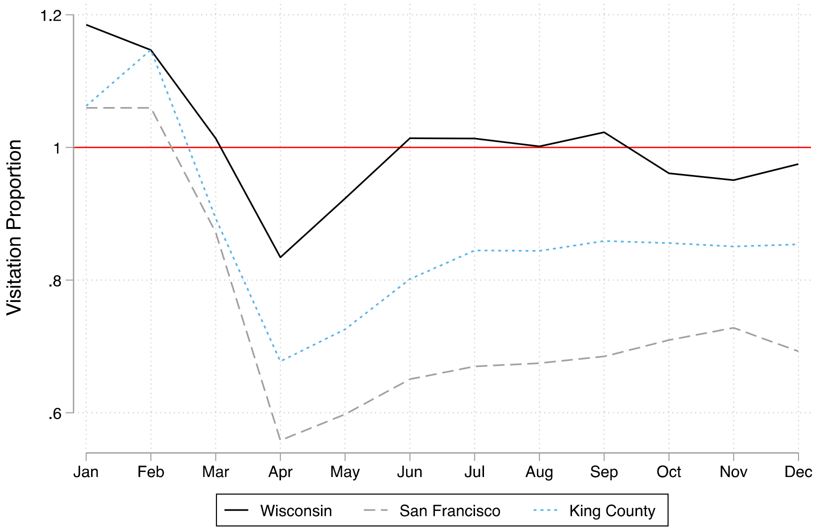

S1-A. Ratio of Mean 2020 Tract Estimated Visitation to 2019 Value

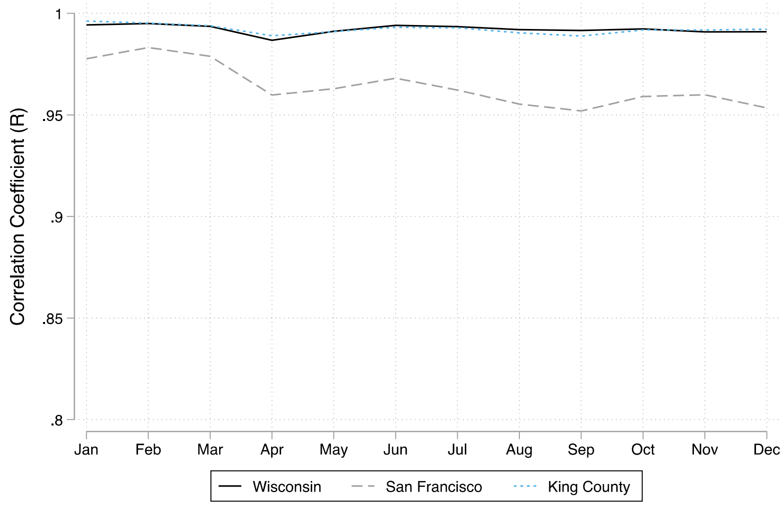

S1-B. Tract Correlation of Same-Month MND Values between 2019 & 2020

Fig. S1. Comparing Same-Month Visitation and MND Levels between 2019 and 2020, by Jurisdiction

Note: In S1-Al, analogously to equation 3 (main manuscript), we calculate visitation as: $\sum_{j=1}^{N} V\left(n_{j,i}\right) * P_j , i \neq j$. In S1-B, we calculate MND values as described in Materials and Methods (main manuscript), using monthly neighborhood network degree values estimated with the single month of SafeGraph data rather than annual data. RND values do not vary.

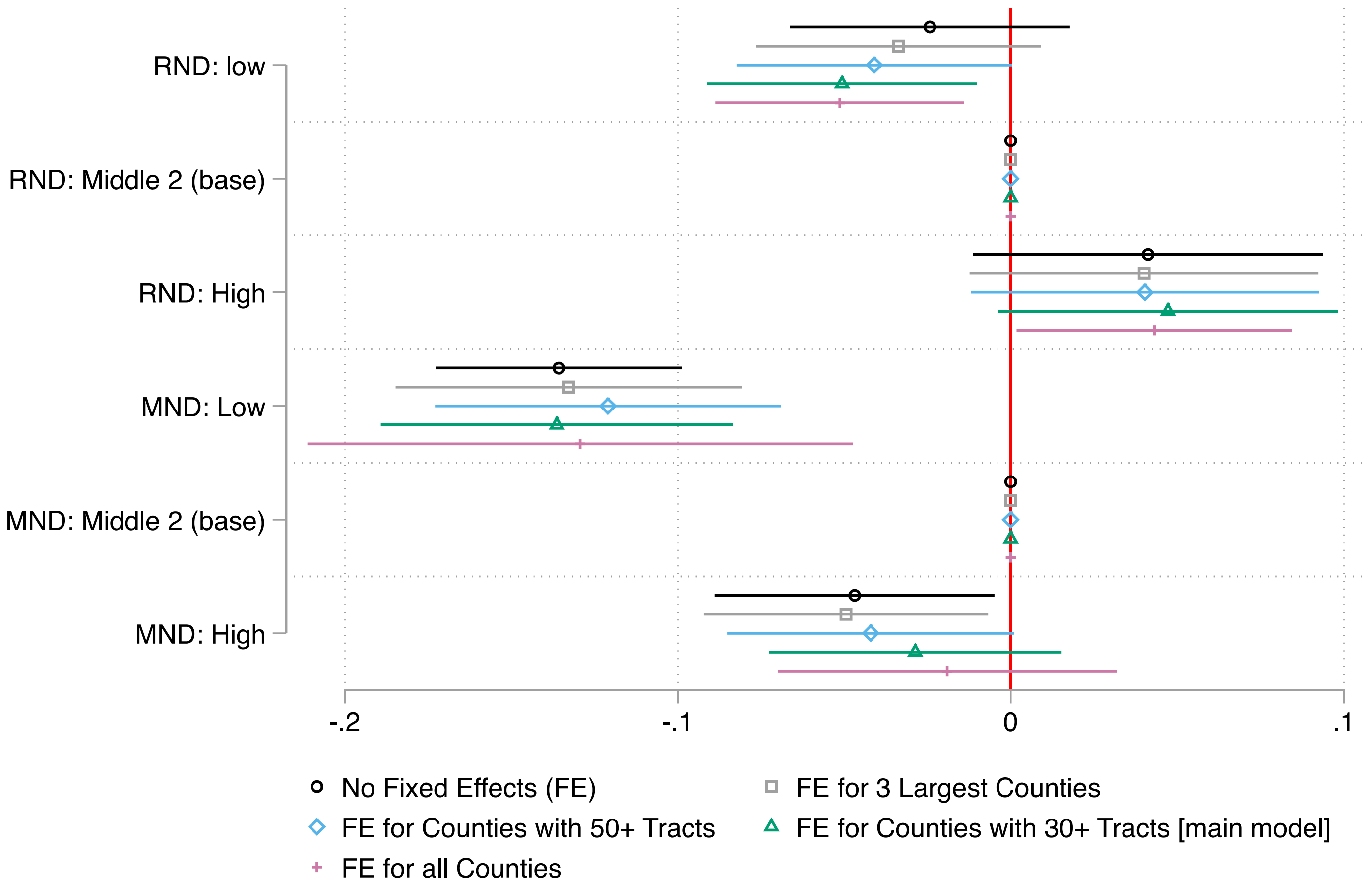

Fig. S2. Sensitivity of Wisconsin Main Model (Manuscript Table 1, Model 6) Estimates of the Relationships between ND Indicators and COVID-19 Case Counts to Alternative Fixed Effects Specifications

Note: All models follow the same specification as manuscript Table 1 Model 6 with the exception of using the alternative specifications of county fixed effects specified in the legend. Figure presents estimated regression coefficients and their 95 percent confidence intervals for the relationship between ND indicators and COVID-19 case counts based Poisson models with robust errors.

	Wisconsin		San Francisco		King County	
	Mean	SD	Mean	SD	Mean	SD
COVID positive	395	212	173	132	200	132
RND	-0.050	0.819	-0.960	0.925	-0.828	0.850
IND	-0.080	0.436	-0.785	0.295	-0.724	0.451
OND	-0.128	0.396	-0.905	0.230	-0.719	0.484
MND	-0.104	0.410	-0.845	0.254	-0.721	0.463
Spatial lag RND	-0.069	0.666	-0.994	0.723	-0.821	0.730
% Black	0.084	0.197	0.053	0.075	0.062	0.076
% Hispanic	0.072	0.115	0.152	0.111	0.096	0.077
Total population	4,162	1,778	4,595	1,852	5,530	1,851
ln(density)	6.526	2.168	10.178	0.670	8.373	1.147
% age 0-4	0.057	0.023	0.046	0.022	0.057	0.021
% age 5-17	0.162	0.050	0.088	0.048	0.146	0.053
% age 18-24	0.094	0.097	0.069	0.071	0.080	0.076
% age 65+	0.167	0.065	0.157	0.067	0.134	0.052
% HH size 4+	0.196	0.070	0.183	0.123	0.226	0.099
HH size	2.404	0.327	2.462	0.651	2.530	0.429
% group quarters	0.024	0.072	0.024	0.066	0.018	0.062
% healthcare	0.042	0.034	0.031	0.030	0.027	0.021
% food prep.	0.058	0.035	0.060	0.047	0.054	0.036
% personal care	0.024	0.016	0.029	0.020	0.028	0.018
% production	0.103	0.054	0.021	0.020	0.037	0.028
% carpool	0.083	0.040	0.071	0.041	0.096	0.042
% public transit	0.024	0.048	0.360	0.089	0.132	0.086

Table S1. Summary Statistics

Note: Sample sizes for Wisconsin, San Francisco, and King County, WA are 1,390, 190, and 397, respectively.

	Mobility-Based ND		
	Low	Middle 2	High
RND			
Low	240	103	5
Middle 2	99	474	122
High	9	118	220

S2-A. Wisconsin (n=1,390)

	Mobility-Based ND		
	Low	Middle 2	High
RND			
Low	33	15	0
Middle 2	15	71	9
High	0	9	38

S2-B. San Francisco (n=190)

	Mobility-Based ND		
	Low	Middle 2	High
RND			
Low	64	36	0
Middle 2	35	138	25
High	1	24	74

S2-C. King County, WA (n=397)

Table S2. Bivariate Frequency Table of RND and Mobility-Based ND Quartiles
Type or paste caption here. Create a page break and paste in the Table above the caption.

	Model 0	Model 1	Model 2	Model 3	Model 4	Model 5	Model 6	Model 7
RND low		-0.030		-0.074***		-0.024	-0.051*	-0.053*
		[0.019]		[0.022]		[0.021]	[0.021]	[0.021]
RND high		0.110***		0.043		0.041	0.047†	0.055*
		[0.025]		[0.028]		[0.027]	[0.026]	[0.026]
MND low		-0.143***			-0.144***	-0.136***	-0.136***	-0.141***
		[0.019]			[0.018]	[0.019]	[0.027]	[0.025]
MND high		-0.059*			-0.040†	-0.047*	-0.029	-0.002
		[0.023]			[0.022]	[0.021]	[0.022]	[0.024]
Spatial lag RND low								0.006
								[0.020]
Spatial lag RND high								-0.056*
								[0.022]
ln(density)			0.016***	0.020***	0.020***	0.021***	0.020***	0.021***
			[0.004]	[0.005]	[0.004]	[0.005]	[0.005]	[0.005]
% age 0-4			-1.830***	-2.055***	-1.984***	-2.087***	-2.199***	-2.178***
			[0.444]	[0.455]	[0.436]	[0.446]	[0.457]	[0.454]
% age 5-17			-1.537***	-1.692***	-1.542***	-1.630***	-1.796***	-1.822***
			[0.409]	[0.408]	[0.398]	[0.400]	[0.425]	[0.425]
% age 18-24			-0.121	-0.224	-0.171	-0.221	-0.255	-0.257
			[0.325]	[0.310]	[0.309]	[0.302]	[0.321]	[0.319]
% age 65+			-0.22	-0.222	-0.232	-0.223	-0.474*	-0.472*
			[0.231]	[0.227]	[0.227]	[0.226]	[0.227]	[0.228]
% HH size 4+			0.464	0.571†	0.616†	0.647†	0.699*	0.701*
			[0.346]	[0.344]	[0.337]	[0.338]	[0.337]	[0.336]
HH size			0.064	0.079	0.06	0.069	0.052	0.053
			[0.064]	[0.064]	[0.062]	[0.062]	[0.063]	[0.063]
% group quarters			0.634*	0.635*	0.608*	0.608*	0.569*	0.567*
			[0.282]	[0.275]	[0.278]	[0.274]	[0.280]	[0.276]
% healthcare			0.282	-0.041	0.018	-0.124	-0.062	-0.013
			[0.388]	[0.412]	[0.393]	[0.413]	[0.396]	[0.393]
% food prep.			0.279	-0.002	0.054	-0.087	-0.335	-0.365
			[0.243]	[0.252]	[0.237]	[0.244]	[0.228]	[0.228]
% personal care			-0.85	-1.044†	-0.886	-0.96	-1.065†	-1.072†
			[0.625]	[0.622]	[0.595]	[0.595]	[0.577]	[0.577]

% production			1.187***	0.858***	0.795***	0.666***	0.404*	0.415**
			[0.150]	[0.160]	[0.142]	[0.156]	[0.158]	[0.157]
% carpool			-0.401†	-0.479*	-0.514*	-0.543*	-0.418†	-0.397†
			[0.219]	[0.222]	[0.220]	[0.223]	[0.219]	[0.221]
% public transit			-1.354***	-1.255**	-1.121**	-1.101**	-0.775*	-0.771*
			[0.398]	[0.395]	[0.387]	[0.386]	[0.362]	[0.359]
% Black			0.027	-0.011	0.027	0.01	-0.051	-0.041
			[0.079]	[0.080]	[0.081]	[0.082]	[0.094]	[0.094]
% Hispanic			0.717***	0.662***	0.739***	0.712***	0.706***	0.729***
			[0.077]	[0.080]	[0.079]	[0.083]	[0.089]	[0.089]
Constant	-2.355***	-2.320***	-2.444***	-2.393***	-2.361***	-2.341***	-2.186***	-2.194***
	[0.008]	[0.012]	[0.139]	[0.136]	[0.136]	[0.135]	[0.131]	[0.131]
Offset: ln(population)	X	X	X	X	X	X	X	X
County F.E.							X	X
BIC	51,580	47,384	39,668	39,284	38,150	38,063	35,292	35,174
Pseudo R^2	0	.0819	0.2332	0.2410	0.2629	0.2649	0.3199	0.3225
RMSE	124.1	116.5	106.5	105.8	102.7	102.6	97.9	97.7

Table S3. Poisson Models of Cumulative Tract COVID-19 Positive Cases through February 2021 (Wisconsin, complete estimates)

***p≤0.001; **p≤0.01; *p≤0.05; †p≤0.1

Note: County fixed effects (F.E.) are dummy variables separately identifying each county with at least thirty tracts. Robust standard errors are in brackets. N=1,390 for all models.

	Model 0	Model 1	Model 2	Model 3	Model 4	Model 5	Model 6
RND low		-0.094		-0.037		-0.02	-0.038
		[0.068]		[0.072]		[0.067]	[0.069]
RND high		0.198†		0.301***		0.169*	0.144*
		[0.120]		[0.079]		[0.071]	[0.068]
MND low		-0.256***			-0.123*	-0.137*	-0.158*
		[0.068]			[0.059]	[0.061]	[0.062]
MND high		0.594***			0.413***	0.362***	0.283***
		[0.119]			[0.069]	[0.070]	[0.079]
Spatial lag RND low							0.013
							[0.067]
Spatial lag RND high							0.159*
							[0.077]
ln(density)			-0.093*	-0.098*	-0.133***	-0.130***	-0.117**
			[0.046]	[0.044]	[0.038]	[0.038]	[0.040]
% age 0-4			-2.909**	-1.782†	-2.327*	-1.807†	-1.807†
			[1.048]	[1.031]	[0.952]	[0.961]	[0.965]
% age 5-17			-2.526***	-2.183**	-2.274**	-2.074**	-1.846*
			[0.764]	[0.788]	[0.723]	[0.726]	[0.718]
% age 18-24			-2.903***	-3.055***	-3.193***	-3.244***	-3.286***
			[0.660]	[0.732]	[0.569]	[0.622]	[0.656]
% age 65+			-0.394	-0.454	-0.574	-0.63	-0.688
			[0.450]	[0.458]	[0.411]	[0.411]	[0.422]
% HH size 4+			0.76	-0.019	-0.049	-0.344	-0.335
			[0.789]	[0.704]	[0.631]	[0.619]	[0.602]
HH size			-0.115	-0.064	-0.069	-0.057	-0.056
			[0.156]	[0.146]	[0.127]	[0.125]	[0.121]
% group quarters			-0.028	0.161	0.528	0.576	0.591
			[0.548]	[0.611]	[0.434]	[0.467]	[0.489]
% healthcare			1.912*	0.546	0.373	-0.237	-0.683
			[0.796]	[0.876]	[0.692]	[0.807]	[0.868]
% food prep.			1.517***	0.778	0.932*	0.528	0.321
			[0.448]	[0.480]	[0.462]	[0.464]	[0.448]
% personal care			1.485	0.623	0.138	-0.216	-0.237
			[1.207]	[1.173]	[1.040]	[1.063]	[1.057]

% production			5.230***	4.633**	4.435***	4.181**	3.659**
			[1.400]	[1.438]	[1.229]	[1.312]	[1.384]
% carpool			0.318	0.745	0.24	0.509	0.388
			[0.905]	[0.899]	[0.824]	[0.807]	[0.799]
% public transit			0.209	0.391	0.487†	0.585*	0.675**
			[0.306]	[0.325]	[0.250]	[0.252]	[0.244]
% Black			2.857***	2.242***	2.295***	2.005***	1.911***
			[0.356]	[0.380]	[0.334]	[0.352]	[0.343]
% Hispanic			3.098***	3.127***	2.757***	2.795***	2.784***
			[0.249]	[0.241]	[0.233]	[0.229]	[0.229]
Constant	-3.278***	-3.480***	-2.725***	-2.733***	-2.246***	-2.297***	-2.422***
	[0.050]	[0.068]	[0.609]	[0.573]	[0.522]	[0.517]	[0.532]
Offset: ln(population)	X	X	X	X	X	X	X
BIC	14,053	8,182	3,769	3,581	3,287	3,243	3,210
Pseudo R^2	0	0.4194	0.7381	0.7522	0.7731	0.7770	0.7801
RMSE	119.6	93.0	51.7	49.8	45.6	45.0	43.8

Table S4. Poisson Models of Cumulative Tract COVID-19 Positive Cases through February 2021 (San Francisco, complete estimates)

***p≤0.001; **p≤0.01; *p≤0.05; †p≤0.1

Note: Robust standard errors are in brackets. N=190 for all models.

	Model 0	Model 1	Model 2	Model 3	Model 4	Model 5	Model 6
RND low		-0.348***		-0.264***		-0.190***	-0.153***
		[0.038]		[0.045]		[0.044]	[0.044]
RND high		0.406***		0.225***		0.124*	0.113*
		[0.047]		[0.058]		[0.059]	[0.057]
MND low		-0.260***			-0.234***	-0.182***	-0.113*
		[0.041]			[0.039]	[0.039]	[0.045]
MND high		0.369***			0.287***	0.253***	0.184**
		[0.048]			[0.045]	[0.048]	[0.060]
Spatial lag RND low							-0.143**
							[0.047]
Spatial lag RND high							0.110†
							[0.060]
ln(density)			0.005	0.016	0.015	0.021	0.023
			[0.021]	[0.020]	[0.018]	[0.018]	[0.019]
% age 0-4			-2.079*	-1.583†	-0.81	-0.762	-0.898
			[1.015]	[0.925]	[0.910]	[0.862]	[0.869]
% age 5-17			-2.464***	-1.991***	-1.355**	-1.216*	-1.322**
			[0.513]	[0.481]	[0.486]	[0.476]	[0.468]
% age 18-24			0.121	-0.159	-0.182	-0.313	-0.309
			[0.401]	[0.406]	[0.398]	[0.407]	[0.401]
% age 65+			0.436	0.176	0.401	0.226	0.087
			[0.361]	[0.350]	[0.315]	[0.315]	[0.316]
% HH size 4+			-0.789	-0.505	-0.867	-0.637	-0.588
			[0.612]	[0.566]	[0.541]	[0.529]	[0.523]
HH size			0.353*	0.245†	0.272*	0.206	0.189
			[0.152]	[0.142]	[0.132]	[0.129]	[0.128]
% group quarters			0.113	0.138	0.147	0.153	0.116
			[0.455]	[0.453]	[0.458]	[0.455]	[0.452]
% healthcare			2.845***	2.145**	2.017**	1.678*	1.644*
			[0.841]	[0.751]	[0.727]	[0.703]	[0.700]
% food prep.			0.071	-0.658	0.77	0.18	-0.013
			[0.595]	[0.584]	[0.550]	[0.562]	[0.564]
% personal care			1.117	0.343	1.481*	0.996	1.01
			[0.713]	[0.648]	[0.696]	[0.662]	[0.658]

% production			5.102***	3.859***	2.870***	2.464***	2.391***
			[0.633]	[0.599]	[0.638]	[0.626]	[0.617]
% carpool			0.192	-0.005	0.009	-0.095	-0.269
			[0.441]	[0.409]	[0.386]	[0.374]	[0.373]
% public transit			-1.325***	-0.989***	-0.829***	-0.677**	-0.658**
			[0.256]	[0.254]	[0.240]	[0.245]	[0.247]
% Black			2.176***	1.530***	1.485***	1.205***	1.129***
			[0.233]	[0.256]	[0.238]	[0.254]	[0.258]
% Hispanic			2.307***	1.749***	1.743***	1.490***	1.452***
			[0.221]	[0.239]	[0.222]	[0.242]	[0.245]
Constant	-3.318***	-3.458***	-4.264***	-3.987***	-4.248***	-4.065***	-3.965***
	[0.030]	[0.030]	[0.362]	[0.340]	[0.308]	[0.308]	[0.311]
Offset: ln(population)	X	X	X	X	X	X	X
BIC	27,018	9,475	9,195	8,568	8,131	7,899	7,788
Pseudo R^2	0	0.6503	0.6634	0.6870	0.7032	0.7122	0.7168
RMSE	119.4	62.4	60.4	56.3	54.4	52.9	52.4

Table S5. Poisson Models of Cumulative Tract COVID-19 Positive Cases through February 2021 (King County, complete estimates)

***p≤0.001; **p≤0.01; *p≤0.05; †p≤0.1

Note: Robust standard errors are in brackets. N=397 for all models.

	WI: Not Milwaukee		WI: Milwaukee	
	Model 1	Model 2	Model 3	Model 4
**Mediator:**	RND	MND	RND	MND
**Main Model (Poisson), Select Parameter Estimates**				
% Black	1.060***	0.813***	-0.079**	-0.163***
	[0.058]	[0.049]	[0.027]	[0.034]
% Black * Mediator	-0.603***	-1.434***	-0.104***	-0.117**
	[0.036]	[0.088]	[0.019]	[0.044]
RND	0.157***		0.145***	
	[0.006]		[0.009]	
Mobility ND		0.290***		0.236***
		[0.009]		[0.022]
**Mediator Model (OLS), Select Parameter Estimates**				
% Black	2.345**	0.748***	1.273	1.043***
	[0.279]	[0.167]	[0.164]	[0.070]
Total Effect	1.185	1.154	0.984	0.988
	(1.069, 1.284)	(1.054, 1.248)	(0.859, 1.129)	(0.872, 1.130)
Net Direct Effect	1.157	1.142	0.928	0.874
	(1.041, 1.260)	(1.032, 1.247)	(0.825, 1.061)	(0.724, 1.112)
Net Indirect Effect	1.024	1.011	1.060	1.131
	(1.009, 1.284)	(0.997, 1.028)	(0.980, 1.177)	(0.941, 1.353)
% Mediated	15.2%	7.8%	-3.4%	-9.2%

Table S6. Mediation Models of the Relationship between Tract Percent Black and Cumulative COVID-19 Positive Cases through February 2021 (Wisconsin)

***p≤0.001; **p≤0.01; *p≤0.05; †p≤0.1

Note: All models (main and mediator) include all control variables, and Wisconsin models include fixed effects for counties with thirty or more tracts. This aligns with our main model (Table S3, Model 6). To calculate total effect and (in)direct effects, contrasts for the % Black variable are at their location-specific 5th and 95th percentiles (0 and 0.122 for non-Milwaukee counties and 0.005 and 0.915 for Milwaukee County). Standard errors are in brackets. For total and (in)direct effects, we bootstrap 95% confidence intervals (in parentheses) using 1,000 replications as recommended by Valeri and Vanderweele (2013). N=1,094 for Models 1-2; n=296 for Models 3-4.

	Model 1	Model 2
**Mediator:**	RND	MND
**Main Model (Poisson), Select Parameter Estimates**		
% Black	2.163***	1.319***
	[0.112]	[0.172]
% Black * Mediator	-0.400***	-0.140
	[0.081]	[0.286]
RND	0.236***	
	[0.020]	
Mobility ND		-1.353***
		[0.055]
**Mediator Model (OLS), Select Parameter Estimates**		
% Black	3.805***	-1.123***
	[0.360]	[0.139]
Total Effect	1.788	1.691
	(1.508, 2.188)	(1.454, 2.004)
Net Direct Effect	1.599	1.295
	(1.312, 2.080)	(1.029, 1.562)
Net Indirect Effect	1.119	1.306
	(0.993, 1.273)	(1.182, 1.591)
% Mediated	24.1%	57.3%

Table S7. Mediation Models of the Relationship between Tract Percent Black and Cumulative COVID-19 Positive Cases through February 2021 (San Francisco)

***p≤0.001; **p≤0.01; *p≤0.05; †p≤0.1

Note: All models (main and mediator) include all control variables. This aligns with our main model (Table S4, Model 5). To calculate total effect and (in)direct effects, contrasts for the % Hispanic variable are at their location-specific 5th and 95th percentiles: 0 and 0.179. Standard errors are in brackets. For total and (in)direct effects, we bootstrap 95% confidence intervals (in parentheses) using 1,000 replications as recommended by Valeri and Vanderweele (2013). N=190.

	Model 1	Model 2
**Mediator:**	RND	MND
**Main Model (Poisson), Select Parameter Estimates**		
% Black	0.553***	0.834***
	[0.068]	[0.076]
% Black * Mediator	-1.347***	-0.960
	[0.066]	[0.152]
RND	0.580***	
	[0.013]	
Mobility ND		-0.096***
		[0.016]
**Mediator Model (OLS), Select Parameter Estimates**		
% Black	3.424***	1.717***
	[0.256]	[0.214]
Total Effect	1.952	1.747
	(1.732, 2.168)	(1.568, 1.938)
Net Direct Effect	1.581	1.468
	(1.315, 1.859)	(1.227, 1.751)
Net Indirect Effect	1.235	1.190
	(1.125, 1.396)	(1.055, 1.340)
% Mediated	39.0%	37.4%

Table S8. Mediation Models of the Relationship between Tract Percent Black and Cumulative COVID-19 Positive Cases through February 2021 (King County)

***p≤0.001; **p≤0.01; *p≤0.05; †p≤0.1

Note: All models (main and mediator) include all control variables. This aligns with our main model (Table S5, Model 5). To calculate total effect and (in)direct effects, contrasts for the % Black variable are at their location-specific 5th and 95th percentiles: 0 and 0.240. Standard errors are in brackets. For total and (in)direct effects, we bootstrap 95% confidence intervals (in parentheses) using 1,000 replications as recommended by Valeri and Vanderweele (2013). N=397.

	WI: Not Milwaukee		WI: Milwaukee	
	Model 1	Model 2	Model 3	Model 4
**Mediator:**	RND	MND	RND	MND
**Main Model (Poisson), Select Parameter Estimates**				
% Hispanic	0.403***	0.186***	0.874***	0.911***
	[0.037]	[0.034]	[0.048]	[0.055]
% Hispanic * Mediator	-0.394***	-1.240***	-0.017	-0.101†
	[0.031]	[0.065]	[0.021]	[0.055]
RND	0.155***		0.123***	
	[0.006]		[0.008]	
Mobility ND		0.317***		0.213***
		[0.009]		[0.019]
**Mediator Model (OLS), Select Parameter Estimates**				
% Hispanic	0.778***	0.073	1.929	1.047***
	[0.205]	[0.123]	[0.228]	[0.097]
Total Effect	1.095	1.071	2.188	2.139
	(1.020, 1.174)	(1.008, 1.136)	(1.952, 2.505)	(1.915, 2.469)
Net Direct Effect	1.083	1.069	1.877	1.925
	(1.001, 1.165)	(1.005, 1.137)	(1.605, 2.203)	(1.612, 2.386)
Net Indirect Effect	1.011	1.001	1.166	1.111
	(1.003, 1.027)	(0.998, 1.012)	(1.049, 1.398)	(0.926, 1.325)
% Mediated	13.0%	2.0%	26.2%	18.8%

Table S9. Mediation Models of the Relationship between Tract Percent Hispanic and Cumulative COVID-19 Positive Cases through February 2021 (Wisconsin)

***p≤0.001; **p≤0.01; *p≤0.05; †p≤0.1

Note: All models (main and mediator) include all control variables, and Wisconsin models include fixed effects for counties with thirty or more tracts. This aligns with our main model (Table S3, Model 6). To calculate total effect and (in)direct effects, contrasts for the % Hispanic variable are at their location-specific 5th and 95th percentiles (0.004 and 0.169 for non-Milwaukee counties and 0.007 and 0.729 for Milwaukee County). Standard errors are in brackets. For total and (in)direct effects, we bootstrap 95% confidence intervals (in parentheses) using 1,000 replications as recommended by Valeri and Vanderweele (2013). N=1,094 for Models 1-2; n=296 for Models 3-4.

	Model 1	Model 2
**Mediator:**	RND	MND
**Main Model (Poisson), Select Parameter Estimates**		
% Hispanic	3.192***	3.418***
	[0.071]	[0.184]
% Hispanic * Mediator	0.669***	1.609***
	[0.070]	[0.248]
RND	0.092***	
	[0.022]	
Mobility ND		1.082***
		[0.066]
**Mediator Model (OLS), Select Parameter Estimates**		
% Hispanic	0.985***	0.672***
	[0.251]	[0.097]
Total Effect	2.744	3.032
	(2.127, 3.254)	(2.428, 3.676)
Net Direct Effect	2.421	2.011
	(1.878, 2.983)	(1.573, 2.474)
Net Indirect Effect	1.133	1.508
	(1.032, 1.279)	(1.290, 1.884)
% Mediated	18.5%	50.2%

Table S10. Mediation Models of the Relationship between Tract Percent Hispanic and Cumulative COVID-19 Positive Cases through February 2021 (San Francisco)

***p≤0.001; **p≤0.01; *p≤0.05; †p≤0.1
Note: All models (main and mediator) include all control variables. This aligns with our main model (Table S4, Model 5). To calculate total effect and (in)direct effects, contrasts for the % Hispanic variable are at their location-specific 5th and 95th percentiles: 0.042 and 0.397. Standard errors are in brackets. For total and (in)direct effects, we bootstrap 95% confidence intervals (in parentheses) using 1,000 replications as recommended by Valeri and Vanderweele (2013). N=190.

	Model 1	Model 2
**Mediator:**	RND	MND
**Main Model (Poisson), Select Parameter Estimates**		
% Hispanic	0.877***	1.204***
	[0.071]	[0.064]
% Hispanic * Mediator	-0.529***	-1.219***
	[0.057]	[0.139]
RND	0.532***	
	[0.013]	
Mobility ND		0.728***
		[0.020]
**Mediator Model (OLS), Select Parameter Estimates**		
% Hispanic	3.491***	1.519***
	[0.261]	[0.217]
Total Effect	1.995	1.993
	(1.724, 2.275)	(1.751, 2.256)
Net Direct Effect	1.425	1.714
	(1.163, 1.707)	(1.456, 2.026)
Net Indirect Effect	1.400	1.162
	(1.274, 1.579)	(1.077, 1.292)
% Mediated	57.3%	28.1%

Table S11. Mediation Models of the Relationship between Tract Percent Hispanic and Cumulative COVID-19 Positive Cases through February 2021 (King County)

***p≤0.001; **p≤0.01; *p≤0.05; †p≤0.1

Note: All models (main and mediator) include all control variables. This aligns with our main model (Table S5, Model 5). To calculate total effect and (in)direct effects, contrasts for the % Hispanic variable are at their location-specific 5th and 95th percentiles: 0.021 and 0.268. Standard errors are in brackets. For total and (in)direct effects, we bootstrap 95% confidence intervals (in parentheses) using 1,000 replications as recommended by Valeri and Vanderweele (2013). N=397.
